# Whole genome sequencing of *Entamoeba nuttalli* reveals mammalian host-related molecular signatures and a novel octapeptide-repeat surface protein

**DOI:** 10.1371/journal.pntd.0007923

**Published:** 2019-12-05

**Authors:** Masayuki Tanaka, Takashi Makiuchi, Tomoyoshi Komiyama, Takashi Shiina, Ken Osaki, Hiroshi Tachibana

**Affiliations:** 1 Department of Bioinformatics, Support Center for Medical Research and Education, Tokai University, Isehara, Kanagawa, Japan; 2 Department of Parasitology, Tokai University School of Medicine, Isehara, Kanagawa, Japan; 3 Department of Clinical Pharmacology, Tokai University School of Medicine, Isehara, Kanagawa, Japan; 4 Department of Molecular Life Science, Tokai University School of Medicine, Isehara, Kanagawa, Japan; 5 Pacific Biosciences Division, Tomy Digital Biology Co., Ltd, Bunkyo-ku, Tokyo, Japan; Temple University, UNITED STATES

## Abstract

The enteric protozoa *Entamoeba histolytica* is the causative agent of amebiasis, which is one of the most common parasitic diseases in developed and developing countries. *Entamoeba nuttalli* is the genetically closest species to *E*. *histolytica* in current phylogenetic analyses of *Entamoeba* species, and is prevalent in wild macaques. Therefore, *E*. *nuttalli* may be a key organism in which to investigate molecules required for infection of human or non-human primates. To explore the molecular signatures of host-parasite interactions, we conducted *de novo* assembly of the *E*. *nuttalli* genome, utilizing self-correction of PacBio long reads and polishing corrected reads using Illumina short reads, followed by comparative genomic analysis with two other mammalian and a reptilian *Entamoeba* species. The final draft assembly of *E*. *nuttalli* included 395 contigs with a total length of approximately 23 Mb, and 9,647 predicted genes, of which 6,940 were conserved with *E*. *histolytica*. In addition, we found an *E*. *histolytica*-specific repeat known as ERE2 in the *E*. *nuttalli* genome. GO-term enrichment analysis of mammalian host-related molecules indicated diversification of transmembrane proteins, including AIG1 family and BspA-like proteins that may be involved in the host-parasite interaction. Furthermore, we identified an *E*. *nuttalli*-specific protein that contained 42 repeats of an octapeptide ([G,E]KPTDTPS). This protein was shown to be localized on the cell surface using immunofluorescence. Since many repeat-containing proteins in parasites play important roles in interactions with host cells, this unique octapeptide repeat-containing protein may be involved in colonization of *E*. *nuttalli* in the intestine of macaques. Overall, our draft assembly provides a valuable resource for studying *Entamoeba* evolution and host-parasite selection.

## Author summary

Determination of host specificity is one of the most significant themes in the field of infectious diseases. Identification of molecules related to the host specificity of pathogens can lead to new treatment and prevention methods, and prediction of potential host shifts. *Entamoeba histolytica* is a human parasite that causes hemorrhagic diarrhea, amebic colitis, and liver abscess, which may result in death in severe cases. *Entamoeba nuttalli* is the closest species to *E*. *histolytica* and infects various wild macaques as natural hosts. *E*. *nuttalli* might also be a pathogen with a zoonotic hazard because severe inflammatory reactions in hamster livers and an asymptomatic case of human infection have been recorded. Here, we report *E*. *nuttalli* genomic data with a quality that allows comparison with genomes from other *Entamoeba* species. Comparative genomics revealed common proteins shared with other mammalian *Entamoeba* species, as well as *E*. *nuttalli*-specific proteins. Therefore, this study identifies candidate molecules required for host specificity and subsequent pathogenicity of *Entamoeba* species.

## Introduction

The genus *Entamoeba* is an anaerobic protozoan lineage consisting of parasitic species that dwell in the digestive tract of various metazoan hosts, with a few species also isolated from the environment [1–4]. In this taxonomic group, *Entamoeba histolytica*, *Entamoeba dispar*, and *Entamoeba invadens* have a common life cycle of an infectious cyst and vegetative trophozoite, but have different virulence potentials and host specificity. *E*. *histolytica* is the causative agent of human amebic colitis and liver abscess, which results in up to 100,000 deaths annually [5]; *E*. *dispar* colonizes the intestine of humans and non-human primates without invasion [6–9]; and *E*. *invadens* is a pathogenic reptilian parasite and a good model organism for the study of encystation: the conversion process from trophozoite to cyst [10,11]. An *E*. *histolytica*-like amoeba that is virulent but genetically different from *E*. *histolytica* has been isolated from rhesus macaques, and revival of the name *Entamoeba nuttalli* was proposed for this amoeba [12]. *E*. *nuttalli*, as the species most closely related to *E*. *histolytica*, has been isolated from various species of wild macaques and captive non-human primates [12–21]. An asymptomatic case of human infection with *E*. *nuttalli* also occurred in a zoo caretaker. Therefore, *E*. *nuttalli* infection may be problematic for the health of non-human primates and may be a zoonotic hazard [22].

Comparative genomics is used in parasitology to identify virulence factors and functional molecules, to carry out evolutionary analyses, and to investigate molecules related to host range [23–27]. In *Entamoeba* species, this approach has identified AIG1 as a novel virulence factor [28], and has established a correlation between genomic diversity and virulence potential [29] and the contribution of repetitive elements to diversification [30]. However, the molecules responsible for host specificity in *Entamoeba* parasites remain to be identified.

The genome assembly of *E*. *histolytica* has the highest quality among *Entamoeba* genomic databases, and a 20 Mb assembly containing 1,496 scaffolds and 8,201 predicted genes has been proposed [31]. Moreover, analyses of the *E*. *histolytica* genome have revealed a high content of AT bases (approximately 75%) and repetitive elements (approximately 19.7%) [30], indicating that it is difficult to reconstruct the complete genome structure using a short-read sequencer only. Reflecting this, the ploidy of *E*. *histolytica* is still uncertain, but may be tetraploid [32]. *Entamoeba* genomic data generated by a long-read sequencer, such as the Pacific BioSciences platform [33], would allow complex genomic regions to be deciphered and provide a more refined assembly for comprehensive comparative genomics.

In this study, we conducted *de novo* assembly of the *E*. *nuttalli* genome and comparative analysis with published sequences of *E*. *histolytica*, *E*. *dispar*, and *E*. *invadens*. We report here mammalian host-related molecular signatures of *Entamoeba* species and an *E*. *nuttalli*-specific octapeptide-repeat surface protein (which we named PTORS). This draft genome of *E*. *nuttalli* aids in understanding of host specificity and evolution of *Entamoeba* species.

## Materials and methods

### Ethics statement

All animal experiments were performed in accord with the Fundamental Guidelines for Proper Conduct of Animal Experiment and Related Activities in Academic Research Institutions under the jurisdiction of the Ministry of Education, Culture, Sports, Science and Technology, Japan, and reviewed and approved by The Institutional Animal Care and Use Committee at Tokai University (Permit Number 185001).

### Preparation of genomic DNA

Trophozoites of *E*. *nuttalli* P19-061405 strain clone 7 were cultured axenically in TYI-S-33 medium [34] supplemented with 15% adult bovine serum (Sigma-Aldrich, St. Louis, MO) at 37°C. Genomic DNA was isolated as previously described [35]. Briefly, nuclei were obtained by centrifugation after cell lysis in 1% Nonidet P-40. The pellet was lysed with 2% sodium dodecyl sulfate and proteinase K. DNA was extracted four times with phenol-chloroform-isoamyl alcohol and then precipitated with ethanol.

### Whole genome sequencing using the PacBio RS system

For single molecule real-time (SMRT) sequencing, genomic DNA of *E*. *nuttalli* was sheared into 10 kb fragments using g-TUBE (Covaris, Woburn, MA) and the quantity and size distribution were measured using a Qubit Fluorometer (Life Technologies/Thermo Fisher Scientific, Palo Alto, CA) and an Agilent 2100 Bioanalyzer DNA12000 kit (Agilent Technologies, Santa Clara, CA). Double-stranded DNA fragments were end-repaired and hairpin adapters were added via blunt end ligation to produce SMRTbell templates using a PacBio DNA Template Prep Kit 2.0. These templates were then treated with exonucleases III and VII to remove failed ligation products and purified with a 0.45× volume of AMPure PB beads. Final SMRTbell libraries were again assessed using the DNA12000 kit (Agilent Technologies). The sequencing primer to polymerase ratio and loading concentration were determined using a PacBio binding calculator. The sequencing primer was annealed to the single-stranded loop of the SMRTbell template, and primer-annealed templates were then bound to DNA polymerase XL (or C2). MagBeads loading was conducted at 4°C for 20 min per the manufacturer’s guidelines, after which MagBeads-bound, polymerase-template complexes were loaded into zero-mode waveguides of SMRT Cells. Sequencing runs were performed with C2 sequencing chemistry with a 120-min movie (35 Cells) or 2 × 55-min (22 Cells) movies. Thus, in total, 57 SMRT Cells were used for sequencing.

### Whole genome sequencing using the Illumina GAIIx system

Genomic DNA of *E*. *nuttalli* was also sequenced on the Illumina GAIIx platform. A paired-end library was prepared from 3 μg of genomic DNA following the protocol of the TruSeq library construction kit (Illumina Inc., San Diego, CA, USA) after fragmentation by sonication under standard conditions (Covaris). Cluster generation and sequencing were undertaken as per the manufacturer’s protocol for paired-end 114 bp sequence reads. The sequencing template was then loaded on one lane of a proprietary flow cell.

### *De novo* assembly of the *E*. *nuttalli* genome

**Fig 1:**
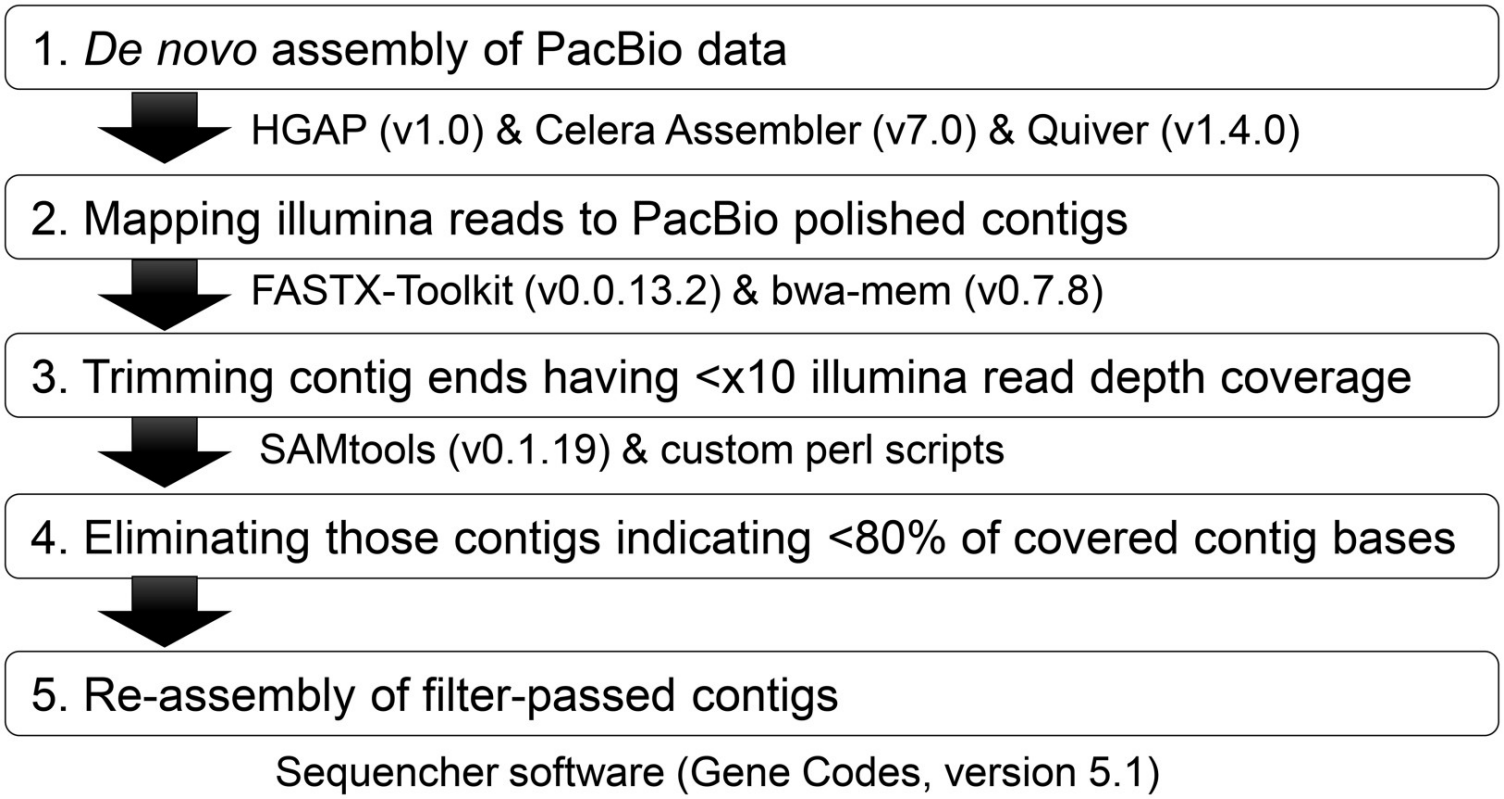
Procedure for reassembly of PacBio and Illumina data.

Long-read and short-read sequence data were utilized in the following procedures (Fig 1). (i) After automatically removing PacBio sub-reads with low accuracy (<80%) and/or short read length (< 500 bases), random errors of sub-reads were corrected by the PreAssembler pipeline of HGAP 1.0 using BLASR [36] with the following parameters: minMatch: 6, -minReadLength: 400, -maxScore: -1200, -bestn: 25, -maxLCPLength: 14, -nCandidates: 50, -allowAdjacentIndels, -indelRate: 0.5. Primary assembly was performed by Celera Assembler 7.0 with an 18 mer size parameter using over 5,000 bases error-corrected sub-reads that had at least 14 times sequence redundancy. The assembly errors within contigs were polished with Quiver [37] in SMRT Analysis v1.4.0. (ii) Raw sequence reads obtained from the Illumina GAIIx platform were quality trimmed to remove poor sequences using FASTX-Toolkit v.0.0.13 with the following parameters: minimum read length (-l): 70 bases, and quality cutoff (-t): 20. The quality-passed reads were mapped to the polished contigs using BWA-MEM v.0.7.8 [38] with default parameters. (iii) Quality assessments and control of poor supportive contigs were then performed with the following criteria: (a) trimming ends of contig with < 10-fold Illumina read coverage of depth, and (b) eliminating contigs with < 4 of 5 covered contig bases. The mapped data were manipulated and alignments were generated in a per-position (pileup) format using SAMtools v.0.1.19 [39]. (iv) The filter-passed contiguous sequences were assembled as >100 bp and 98% matched parameters using Sequencher v.5.1 DNA sequence assembly software (Gene Code Co., Ann Arbor, MI).

### Assessment of genome assemblies

BUSCO v.3.0.2 [40] was utilized to assess the quality of genome assembly. BUSCO analysis used NCBI BLAST+ v.2.6.0, HMMER v.3.1b1 [41] and Augustus v.3.3.1 [42]. BUSCO was run with the following parameters: -l eukaryota_odb9, -m genome. *E*. *histolytica* HM-1:IMSS v.4.0 and *E*. *nuttalli* P19 4.0 were downloaded from AmoebaDB [43].

### *E*. *nuttalli* genome annotation

Gene prediction was performed by GlimmerHMM with a training set as an *E*. *histolytica* gene model. Annotation of the predicted genes was performed by NCBI BLAST against the *E*. *histolytica* protein dataset (AmoebaDB v.4.0) and the NCBI non-redundant protein database (downloaded Jan 30, 2013). Protein signatures, motifs and Gene Ontology annotations were assigned using InterProScan v.5.6–48 [44]. Non-annotated and short amino acid sequences (< 50 amino acids) were removed from further analyses. Finally, predicted genes with overlap with repeat elements were excluded from the annotation after masking of the repeat sequences against the *Entamoeba* genus using RepeatMasker v.4.0.5. (http://www.repeatmasker.org/).

### Search for orthologous clusters across multiple species

The protein dataset of *E*. *invadens* IP-1 v.4.0, *E*. *histolytica* HM-1:IMSS v.4.0, and *E*. *dispar* SAW760 v.4.0 were downloaded from AmoebaDB. The OrthoVenn server [45] was used for identification of mammalian host-related orthologous clusters with *E*. *invadens* IP-1 and *E*. *histolytica* HM-1:IMSS. The DAVID web server [46] was used for GO-term enrichment analysis with Benjamini adjusted P values < 0.05.

### Prediction of cell surface proteins and subcellular localization

Cell surface proteins were predicted *in silico* using SOSUI [47], TMHMM [48,49], and SignalP [50]. Putative transmembrane helix-containing proteins were defined as those indicated by SOSUI as membrane proteins and those in which TMHMM predicted at least one transmembrane helix. Putative signal peptide-containing proteins were defined as those predicted by SignalP to include a signal peptide at the default D-cutoff. WoLF-PSORT [51] was used to predict subcellular localization, with the organism parameter as animal and fungi. WoLF-PSORT annotation with concordance between animal and fungi parameters was used.

### Plasmid construction

To generate recombinant histidine-tagged PTORS without a putative signal peptide (rPTORS), *PTORS* gene was PCR-amplified from template *E*. *nuttalli* genomic DNA using sense (5′-GTC GCA TAT GAT TCT TTG TAT GGA ACA AGG AGT TAA AG-3′) and antisense (5′-GTC GTC TAG ATT AGA AGT AGA TAA ATG CAA TAA CAA TTG-3′) primers. *Nde* I and *Xba* I restriction sites are underlined in the respective primers. The PCR fragment and pCold I plasmid (TaKaRa, Otsu, Japan) were digested by *Nde* I and *Xba* I, and the digested products were ligated using a Ligation-Convenience Kit (Nippongene, Tokyo, Japan). After ligation, the plasmid was transformed in Competent Quick DH5α (Toyobo, Osaka, Japan) and the amplified plasmid was purified using a QIAprep Spin Miniprep Kit (Qiagen GmbH, Hilden, Germany).

### Recombinant protein

The plasmid was transformed into BL21 (DE3) One Shot Chemically Competent *E*. *coli* (Life Technologies) and expression of rPTORS was induced by low temperature (15°C) with 1 mM IPTG. After induction, rPTORS was purified from bacterial lysates using a Ni-NTA system (Qiagen) under denaturing conditions with 6 M urea.

### Antisera

Six-week-old male BALB/c mice were purchased from CLEA Japan, Inc. (Tokyo, Japan). Five mice were immunized subcutaneously with 100 μg of rPTORS emulsified in TiterMax Gold (TiterMax USA, Norcross, GA). Immunization was repeated twice at two-week intervals. Four weeks after the last injection, sera of mice were collected.

### Immunoblot analysis

Immunoblot analysis was performed as previously described [52]. Briefly, antisera for PTORS were diluted 200-fold with PBST containing 5% skim milk (Wako, Osaka, Japan), and anti-mouse immunoglobulin F(ab′)_2_ fragment conjugated with horseradish peroxidase (Amersham) was diluted 3000-fold with PBST.

### Immunofluorescence microscopy

Sample preparation for immunofluorescence microscopy was performed as previously described [53]. Briefly, antisera for PTORS were diluted 100-fold with 3% bovine serum albumin in PBS and Alexa Fluor 488 goat anti-mouse IgG (Life Technologies) was diluted 300-fold with PBS. Confocal fluorescence images were captured using a LSM880 confocal microscope (Carl Zeiss, Jena, Germany) in channel mode and analyzed with ZEN2 software.

## Results

### Sequence read information and draft genome assembly process

Sequence read information was initially obtained by single molecule real-time (SMRT) sequencing of *E*. *nuttalli* genomic DNA using the PacBio RS platform with 57 SMRT Cells. An initial filtering removed all subreads with low accuracy (< 80%) or short read length (< 500 bases), generating 1,094,547 subreads and 2,587,786,482 bases (subread length distributions are shown in S1 Fig). After random errors of subreads were corrected by PreAssembler pipeline in HGAP 1.0 using BLASR, a primary assembly of 1,172 contigs was constructed using Celera Assembler 7.0. Assembly errors within contigs were polished by Quiver in SMRT Analysis v1.4.0, resulting in a total assembly size of 32,595,857 bases, N50 size of 45,298 bases and the longest size of 306,099 bases (step 1 in Fig 1 and Table 1). Before reassembly of the primary assembly, short-reads obtained via the Illumina system were utilized for improvement of primary assembly completeness. Short-read sequencing of the *E*. *nuttalli* genomic DNA produced 36,954,093 paired-end 114-bp reads corresponding to >8,425,533,204 bases. Of 35,088,246 quality-passed paired-end reads, 34,921,637 were mapped to the primary assembly using bwa-mem v.0.7.8 with default parameters (step 2 in Fig 1). The mean read depth coverage of mapped data of the primary assembly was 238.83× coverage, and the percentages of contig sequence coverage above 1×, 50× and 100× sequenced were 91.7, 86.1 and 79.4%, respectively (S1 Table). The primary assembly was supported by most Illumina short-reads, but some 5′ and 3′ contig ends, which are approximately 10% of the sequence from each contig end, clearly had a lower read depth coverage (S2 Fig, upper panel). Therefore, we performed quality control filtration of the primary assembly using the Illumina mapped data, as shown in steps 3 and 4 of Fig 1 (detailed thresholds are given in the Methods). Most 5′ and 3′ contig ends were improved by the quality control filtration (S2 Fig, lower panel). Of 1,172 contigs, 743 that were quality-passed were reassembled using Sequencher v.5.1 with parameters of similarity = 98% and overlap = 100 bp. Finally, a total of 395 contigs (174 assembled and 221 singleton contigs) were obtained as a semi-hybrid assembly. The longest contig was 448,959 bases and N50 was 90,004 bases (Table 1).

**Table 1:** Genome assembly in *E*. *nuttalli* and comparison with public genome assembly.

Item	Primary assembly^a^	Semi-hybrid assembly			Public genome assembly^b^	
		Assembled	Singleton	Total	*E*. *nuttalli*	*E*. *histolytica*
**Number of contigs**	1,172	174	221	395	5,233	1,496
**Number of bases**	32,595,857	13,591,796	9,619,065	23,210,861	14,399,953	20,799,072
**GC Content (%)**	24.19	24.20	24.28	24.24	25.02	24.28
**Mean contig size**	27,812	78,113	43,525	58,761	2,751	13,903
**N50 contig size**	45,298	100,804	75,353	90,004	7,707	49,118
**Longest contig size**	306,099	448,959	215,923	448,959	97,841	530,629
**Shortest contig size**	3,527	7,290	3,175	3,175	124	235
**BUSCO score (%)**^c^	12.2	-	-	48.5	46.6	53.1

^a^ Genome assembly of PacBio data with an adapted assembly error correction using Quiver.

^b^ *E*. *nuttalli* P19-061405 and *E*. *histolytica* HM-1:IMSS downloaded from AmoebaDB v.4.0.

^c^ BUSCO score represented by the percentage classified as ‘Complete’.

The results of the new draft assembly of *E*. *nuttalli* showed similar metrics in GC content and estimated genome size to the current assembly of *E*. *histolytica*. The BUSCO algorithm was used for quality control assessment of the new draft genome assembly, in comparison to the primary assembly and public assembly (Table 1). Coverage of core protein hits was at a similar level to that in the well-annotated genome of *E*. *histolytica*. It is difficult to use BUSCO results as a measure of genomic completeness due to low coverage of core protein hits, but the reassembly process improved the quality of the primary assembly in terms of gene content.

### Characteristics of the *E*. *nuttalli* genome

**Fig 2:**
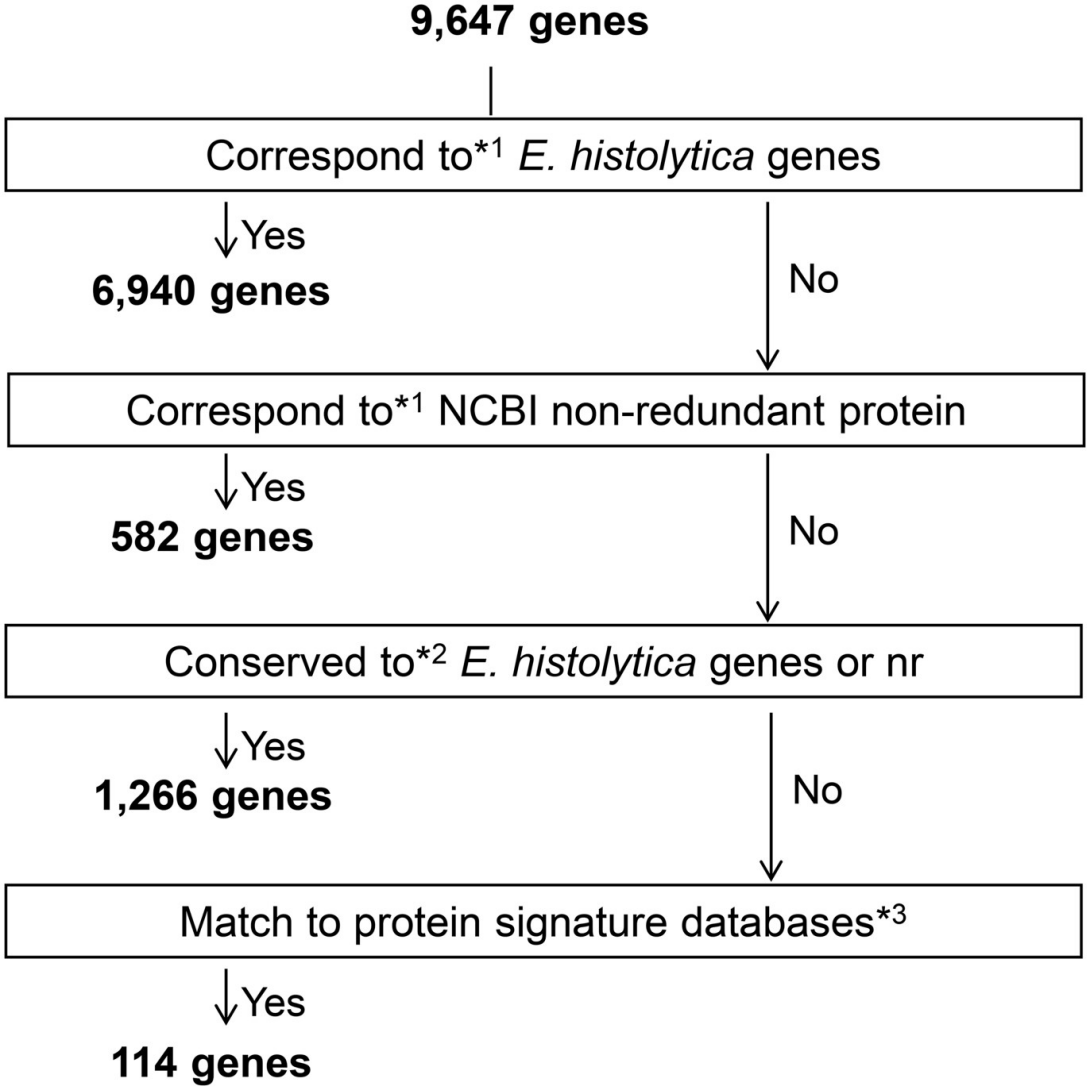
Classification of *E*. *nuttalli* annotated genes. Steps followed in categorization using AmoebaDB, the NCBI non-redundant protein database, and the InterProScan database. Categorization criteria for BLAST results were correspondence defined by blastp using coverage of ≥80% identity, 90% query coverage, and e-value < 1e-10; conservation defined by blastp using coverage of ≥50% identity, 70% query coverage and e-value < 1e-10; and InterProScan with preconfigured thresholds.

To assess the *E*. *nuttalli* genome features, we annotated the 9,647 predicted genes derived from the new assembly and compared the data with genome statistics of *E*. *histolytica*, *E*. *dispar* and *E*. *invadens* in AmoebaDB (Table 2). The results showed that the *E*. *nuttalli* genome had similar metrics to those of the mammalian *Entamoeba* species, including the percentage of coding regions and GC content, despite the larger total genome size and number of annotated genes compared to *E*. *histolytica*. Annotated genes were then classified into the following subsets: 6,940 and 582 genes with best sequence similarity to *E*. *histolytica* proteins and NCBI non-redundant proteins, respectively (≥80% identity, 90% coverage, e-value < 1×10^−10^); 1,266 genes with better sequence similarity to *E*. *histolytica* proteins or NCBI non-redundant proteins (≥50% identity, 70% coverage, e-value < 1×10^−10^); 114 genes assigned with the InterPro collection database and with poor sequence similarity (<50% identity, 70% coverage, and/or e-value > 1×10^−10^); and 745 genes that could not be assigned at the given thresholds (Fig 2).

**Table 2:** Comparison of genome statistics among four *Entamoeba* species.

Item	*E*. *nuttalli*	*E*. histolytica*	*E*. dispar*	*E*. invadens*
**Genome**				
Size (bp)	23,210,861	20,799,072	22,955,291	40,888,805
Number of genes	9,647	8,333	10,262	11,975
GC content (%)	24.23	24.20	23.54	29.92
Mean gene length (bp)	1,189.32	1,283.56	1,084.60	1,354.16
Percent coding (%)	49.43	51.43	48.49	39.66
**Exons**				
Number	12,727	10,891	13,932	17,958
Mean number per gene	1.32	1.31	1.36	1.50
GC content (%)	28.10	27.99	28.00	38.67
Mean length (bp)	880.05	965.15	777.99	869.2
Total length (bp)	11,200,448	10,511,492	10,838,988	15,609,079

* Numbers are data in AmoebaDB v.4.0.

The repeat structure of the assembly was assessed using RepeatMasker software with the *Entamoeba* genus dataset (database 20140131 update). Approximately 21.57% of the genome assembly was classified as repetitive, comprising 5 Mb of DNA sequences. Transposable elements (TEs), such as short and long interspersed nuclear elements (SINEs and LINEs) [54] and *Entamoeba*-specific TEs (ERE1 and ERE2) [30] were also present (Table 3).

**Table 3:** Repeat elements in the *E*. *nuttalli* genome assembly identified by RepeatMasker.

Name	Class/family	Number of sites	Total length (bp)	%Genome*
EHAPT2	SINE	343	139,869	0.60
REP-1_ED	SINE	157	65,024	0.28
EdSINE1	SINE	134	29,462	0.13
R4-1_ED	LINE	805	1,002,819	4.32
EhRLE3	LINE	441	577,331	2.49
R4-N1_ED	LINE	186	81,389	0.35
EhRLE2	LINE	74	106,274	0.46
ERE1_EH	Unknown	960	1,220,105	5.26
ERE2_EH	Unknown	594	718,392	3.10
EHINV1	Unknown	224	71,907	0.31
ERE1_ED	Unknown	101	42,317	0.18
EHINV2	Unknown	68	10,226	0.04
ERE1_EI	Unknown	1	173	< 0.01
SCAI_EH	Repeat_region	977	113,109	0.49
LEAPFROG1_EI	DNA/PiggyBac	1	108	< 0.01
Mogwai2_EI	DNA/TcMar-Mogwai	1	93	< 0.01
Low_complexity	-	3,832	231,577	1.00
Simple_repeat	-	10,618	597,899	2.58
**Total**		**19,517**	**5,008,074**	**21.57**

* Expressed as a percentage of the assembly sequence length

Since surface-exposed proteins of parasites play an important role in the host-pathogen interaction, it is valuable to make a list of such proteins. An *in silico* analysis performed to classify the *E*. *nuttalli* annotated genes predicted that 2,070, 879, 237, and 500 genes coded for transmembrane-containing, signal peptide-containing, extracellular, and plasma membrane proteins, respectively, from the total of 9,647 genes in the *E*. *nuttalli* genome.

### Identification of orthologous clusters among mammalian *Entamoeba* species

**Fig 3:**
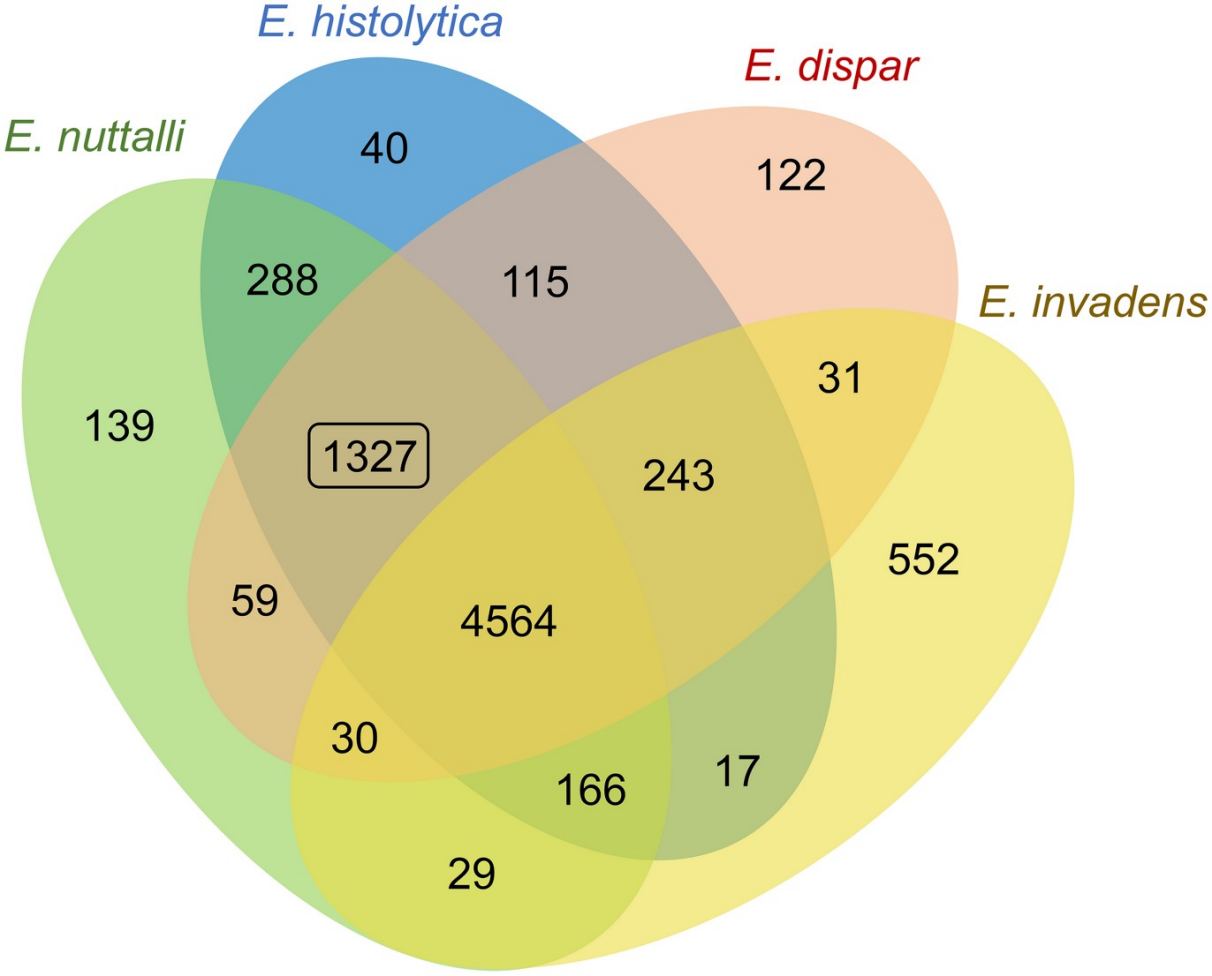
Characterization of mammalian host-related orthologous clusters. Venn diagram of the orthologous gene family in four *Entamoeba* species. The rectangle indicates a possible mammalian host-related orthologous cluster.

To identify specific and common orthologous genome clusters among *E*. *nuttalli*, *E*. *histolytica*, *E*. *dispar*, and *E*. *invadens*, comparative genome analysis was conducted using the OrthoVenn web server with default parameters. The 9,647 predicted proteins in the *E*. *nuttalli* genome were grouped into 6,602 clusters, of which 4,564 were shared with all other *Entamoeba* genomes and 1,327 were shared only with mammalian parasites, *E*. *histolytica* and *E*. *dispar* (Fig 3, S2 Table). To characterize the orthologous proteins shared among these mammalian *Entamoeba* species, GO-term enrichment analysis was performed using the DAVID web server. The 1,327 orthologous clusters comprised 1,591, 1,475, and 1,514 proteins of *E*. *nuttalli*, *E*. *histolytica*, and *E*. *dispar*, respectively. The *E*. *histolytica* orthologous proteins were used as a DAVID query because the *E*. *histolytica* genome is the most curated among *Entamoeba* species. GO-term enrichment analysis of the 1,475 *E*. *histolytica* proteins identified integral membrane components (cellular component, GO:0016021), including six AIG1 family proteins (EHI_022500, EHI_115160, EHI_144270, EHI_089670, EHI_195260 and EHI_195250) and 16 leucine rich repeat proteins as BspA-like proteins (EHI_062750, EHI_192600, EHI_139430, EHI_139390, EHI_054160, EHI_129870, EHI_110760, EHI_137910, EHI_013940, EHI_192250, EHI_082060, EHI_094080, EHI_020090, EHI_100700, EHI_147680 and EHI_127100) with an adjusted P-value < 0.05 (Table 4).

**Table 4:** Enrichment of GO terms for ortholog clusters in mammalian *Entamoeba* species.

GO ID	GO term	GO category	%Gene counts	Corrected P values*
GO:0016021	Integral component of membrane	Cellular component	12.8	0.0026626
GO:0005525	GTP binding	Molecular function	4.0	0.0225842
GO:0007264	Small GTPase mediated signal transduction	Biological process	3.6	0.0023422
GO:0005643	Nuclear pore	Cellular component	0.8	0.0051100
GO:0016788	Hydrolase activity, acting on ester bonds	Molecular function	0.5	0.0131763

* DAVID web server with adjusted P values < 0.05.

### Refinement of candidates for *E*. *nuttalli*-specific surface proteins

Surface-exposed proteins are candidates for adhesion to host cells and defense or evasion from host immune attacks, while species-specific molecules may play an important role in host-specificity. A total of 114 annotated genes in the *E*. *nuttalli* genome had weak matches against a public database and were annotated only in the InterPro database (S3 Fig). Of these *E*. *nuttalli*-specific genes, three (ID; EN0317G0042, EN0144G0007 and EN0096G0007) were predicted to code for extracellular or plasma membrane proteins by WoLF-PSORT.

To examine whether these genes encode species-specific surface molecules, we manually curated the genes. In InterPro annotation, EN0317G0042 was assigned as a Sys1-family protein that functions in protein trafficking between the late Golgi and endosome [55]. Therefore, this protein was excluded from the list of surface-exposed proteins. The predicted amino acid sequence of EN0144G0007 had 94% sequence identity with *E*. *histolytica* SAPLIP6 (EAL50434), which is in the saposin-like protein family [56]. Sequence alignment of SAPLIP6 showed a conserved signal peptide and saposin-like structure (IPR011001) predicted by InterProScan (S4 Fig). A search in AmoebaDB (release 40, 15 Oct 2018) revealed no identical sequences with *E*. *histolytica* SAPLIP6. Therefore, EN0144G0007 was excluded from further analysis.

In contrast to EN0317G0042 and EN0144G0007 proteins, EN0096G0007 was not identified in other amoebozoan organisms, eukaryotes, archaea and bacteria, although some proteins in *E*. *histolytica* had <49% sequence identity with partial regions of EN0096G0007. Phylogenetic reconstruction of EN0096G0007 and its homologs in *Entamoeba* species showed that EN0096G0007 forms an isolated cluster from clusters of putative *E*. *histolytica* homologs, with strong bootstrap support (S5 Fig). Thus, EN0096G0007 may be a species-specific gene, and we conducted further characterization of its features *in silico* and *in vitro*.

### A novel repeat protein specific for *E*. *nuttalli*

**Fig 4:**
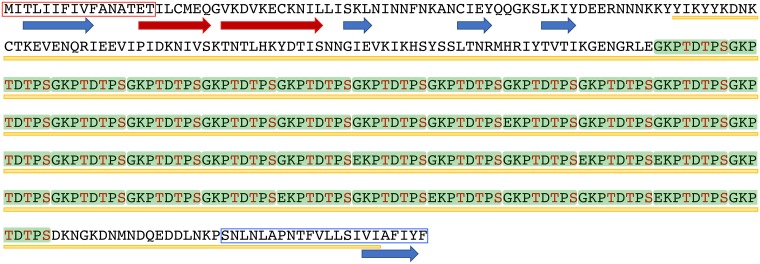
Secondary structure prediction for PTORS. The predicted amino acid sequence of PTORS was subjected to secondary structure analysis by PSIPRED. This is shown as blue arrows for beta strands, red arrows for alpha helices, and yellow lines for disordered regions. For comparison, secondary structure prediction was also performed using Jpred (S6 Fig). A predicted signal peptide sequence (SignalP, red rectangle) and a transmembrane helix (SOSUI and TMPred, blue rectangle) are also shown. Octa-peptide repeat units of PTORS are shown as green rectangles.

The function of EN0096G0007 was predicted using *in silico* analysis of the primary structure (Fig 4). The most remarkable feature of EN0096G0007 is the presence of 42 repeats of an octapeptide (NH_2_-[G,E]KPTDTPS-CO_2_H). Based on the primary structure prediction, we designated EN0096G0007 as PTORS (Proline and Threonine-rich Octapeptide-Repeat Surface protein). This repeat unit contains two threonines and one serine that were predicted to be phosphorylation and/or O-glycosylation sites by NetPhos 3.1 [57] and DictyOGlyc 1.1 [58]. Therefore, PTORS has 126 sites for putative modification in the octapeptide repeat region.

In a secondary structure prediction, a putative signal peptide was detected by SignalP, and two α-helices, five β-strands, and 85.5% disordered regions were predicted by PSIPRED [59]. Almost all the α-helices and β-strands were within 80 residues from the N-terminus. In contrast, a transmembrane α-helix (NH_2_-SNLNLAPNTFVLLSIVIAFIYF-CO_2_H) near the C-terminus was predicted by SOSUI and TMPred (https://embnet.vital-it.ch/software/TMPRED_form.html), but not by TMHMM. GPI-anchor, myristoylation, and prenylation modifications of PTORS were not supported by PredGPI [60] NMT (http://mendel.imp.ac.at/myristate/SUPLpredictor.htm), and PrePS [61], respectively. However, Cys19 and Cys30 of PTORS were predicted as palmitoylation sites by CSS-Palm [62].

### Expression and localization of the novel *E*. *nuttalli*-specific repeat protein

**Fig 5:**
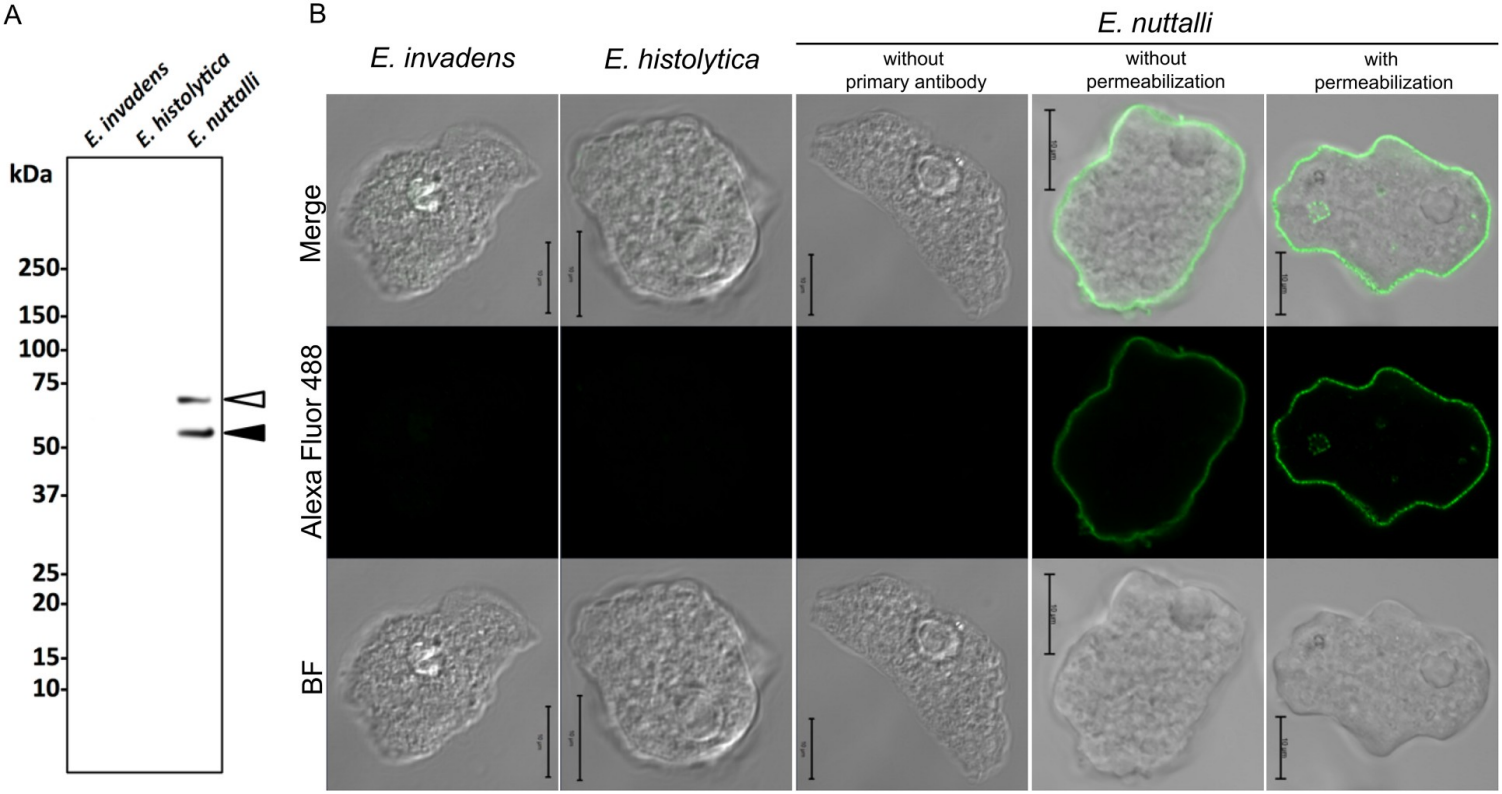
PTORS expression in *Entamoeba* species. (A) Confirmation of expression of PTORS by immunoblot analysis with antisera specific for PTORS. Lanes contain 30 μg protein of whole cell lysates from *E*. *nuttalli*, *E*. *histolytica* HM-1:IMSS, and *E*. *invadens* IP-1. Closed and open arrowheads indicate PTORS bands without and with putative post-translational modification, respectively. (B) Immunofluorescence images of *E*. *nuttalli*, *E*. *histolytica*, and *E*. *invadens* using antisera specific for PTORS. Scale bar = 10 μm. *Entamoeba* species were stained by treatment with/without Triton X-100 and with/without antisera specific for PTORS. Merged bright field (BF) and fluorescence images are also shown (Merge). These images are approximately 0.9 μm in thickness per section.

To confirm the localization of PTORS in *E*. *nuttalli*, we conducted immunoanalyses with murine antisera to recombinant PTORS. An immunoblot analysis using whole cell lysate of *E*. *nuttalli* showed a major band near the expected molecular mass of PTORS (55 kDa) (Fig 5), indicating that the antisera were reactive with a native PTORS. Another major band at approximately 70 kDa might have been due to phosphorylated and/or O-glycosylated PTORS modified post-translationally. Immunofluorescence staining using several *Entamoeba* species and antisera to PTORS (Fig 5 and S7 Fig) revealed fluorescence signals of PTORS on the surface of *E*. *nuttalli* trophozoites treated with Triton X-100. A similar result was observed using *E*. *nuttalli* treated without Triton X-100, strongly suggesting that most PTORS is exposed extracellularly. In addition, *E*. *histolytica*, *E*. *invadens*, *E*. *dispar* and *E*. *moshkovskii* had no proteins that were recognized by antisera to PTORS. These results indicate that PTORS is localized on the plasma membrane of *E*. *nuttalli*.

## Discussion

There are five important outcomes of this study: 1) a new gene catalog and contigs of *E*. *nuttalli* with a more refined assembly, 2) identification of key features of the *E*. *nuttalli* genome, 3) a new comparative analysis among *Entamoeba* genomes, 4) a list of candidate molecules associated with infection of mammalian hosts by *Entamoeba* species, and 5) identification of an *E*. *nuttalli*-specific surface protein that has not been found in other organisms.

Hybrid *de novo* genome assembly was useful for construction of a near-complete genome, especially using the SMRT sequencing platform combined with short-read sequencing in eukaryotic genomes [63–65]. We generated a good quality draft assembly of the *E*. *nuttalli* genome through self-correction of long reads and by polishing the corrected reads using short reads. The new genome assembly is much more complete than the public genome assembly of *E*. *nuttalli* as illustrated by the genomic properties shown in Table 1. Moreover, proteome coverage of our dataset indicated 5,594 orthologous clusters shared with the public dataset of *E*. *nuttalli* and only three orthologous clusters containing six protein sequences that were not included (S8 Fig). Although four of these six sequences were found in our dataset by BLAST search (e-value<1e-10), some of the contigs in the new genome assembly may include uncorrected bases due to sequence errors derived from SMART sequencing. However, the new assembly had an AT content and gene density in *E*. *nuttalli* similar to those in genomes of other *Entamoeba* species. This shows that our annotation dataset is comparable to other *Entamoeba* genomes.

The present analysis also confirms previous reports showing that *E*. *nuttalli* is the closest species to *E*. *histolytica*; indeed, approximately 70% of the *E*. *nuttalli* annotated genes were conserved in *E*. *histolytica* [66,67]. The repeat fraction of the *E*. *nuttalli* genome (21.57%) was more similar to that in *E*. *histolytica* (19.7%) compared to *E*. *dispar* (9.7%) and *E*. *invadens* (9.9%) [30]. Moreover, the population of repeat elements of *E*. *nuttalli* is enriched with non-LTR retroelements, in correspondence with those of other mammalian *Entamoeba* species, while those in non-mammalian species are enriched in class II transposons [30,68–70]. Notably, ERE2, an *E*. *histolytica*-specific repeat, was also found in the *E*. *nuttalli* genome [30], which also supports *E*. *nuttalli* as the closest species to *E*. *histolytica*. Since previous reports have demonstrated that chromosome rearrangements driven by transposable elements contribute to host adaptation [71,72], our findings also suggest that transposable elements have contributed to diversification of *Entamoeba* species, as well as to host adaptation. Nevertheless, we could not achieve chromosome-level sequence assembly. This result suggests that our dataset could not resolve genome complexity such as repetitive elements larger than SMRT sequencing reads and/or low complexity regions leading to misalignment of assembly [73]. Some of those complex regions might contribute to transcriptional silencing of the amoebapore gene [70,74] and diversification of gene families such as light and intermediate subunits of the galactose and N-acetyl-D-galactosamine-inhibitable adherence lectin [75]. It may be possible to improve the assembly utilizing methodologies such as Hi-C and the BioNano Genomics Irys System [76–79].

Our GO-term enrichment analysis revealed a high content of transmembrane components, such as AIG1 family proteins and BspA-like proteins, in the mammalian *Entamoeba* genomes (Table 4). An AIG1 family protein (EHI_176590) has recently been described as a virulence factor that was absent in an *E*. *histolytica* KU27 strain isolated from an asymptomatic cyst passer [28]. Moreover, the *E*. *histolytica* BspA-like protein (EHI_016490) seems to function as a chemoattractant receptor for tumor necrosis factor [80], and bacterial BspA-like proteins are involved in adherence, invasion of epithelial cells, and binding to fibronectin and fibrinogen [81–83]. Interestingly, a recent report has demonstrated that AIG1 and BspA families are undergoing lineage-specific expansion in *E*. *histolytica* [84]. These reports suggest that mammalian *Entamoeba* species have expanded the gene number for cell surface proteins to adapt to different environments in the host digestive tract and/or to develop a virulence mechanism for each host. This may also be supported by our data showing a correlation between the number of pathogenic *Entamoeba* genes for the BspA-like protein and host genes for fibronectin and fibrinogen (S9 Fig). Further analyses of these protein families might reveal the core set of surface proteins required for infection of a mammalian host. Incidentally, our phylogenetic analysis of the *Entamoeba* AIG1 protein family (S10 Fig) showed that *E*. *histolytica* AIG1 (EHI_176590) clustered with proteins of other *Entamoeba* species with moderate bootstrap support, suggesting that the quality, rather than the existence, of AIG1 is important for virulence associated with EHI_176590.

Repeat-containing proteins in intracellular parasites seem to contain a larger number of N- and O-glycosylation sites than those in extracellular parasites. In addition, extracellular parasites tend to contain degenerate repetitive motifs compared with intracellular parasites [85]. Therefore, PTORS found in the extracellular parasite in this study may be an exception because this protein has a large number of putative O-glycosylation sites and almost perfect repetitive motifs (Fig 4). The most important question is the function of this novel surface protein in *E*. *nuttalli*. Recently, O-glycosylated proteins on pathogens and tumors have been reported to contribute to immune evasion [86–88]. Moreover, repeat-containing proteins in parasites play important roles in interactions with host cells, such as adhesion, invasion, virulence, and evasion from the host immune system [89–94]. These reports suggest that *E*. *nuttalli* uses PTORS for evasion of the immune system of the host macaque. Infections with *E*. *nuttalli* have been observed in various species of wild macaques, but host macaques are asymptomatic, indicating a commensal host-parasite relationship in these natural hosts [13,15,17–19]. In contrast, fatal cases of liver abscess with *E*. *nuttalli* have been reported in an Abyssinian colobus and Geoffroy’s spider monkey in a zoo [95,96]; and severe inflammatory reactions have been found in livers of hamsters inoculated experimentally with *E*. *nuttalli*, which indicates pathogenicity for these host species [12,15,17,97]. These findings suggest that the surface molecules of *E*. *nuttalli* evolved to permit colonization in the intestine of natural hosts by keeping a balance with host immunity. The *E*. *nuttalli*-specific protein identified in this study may have an important role in this phenomenon. However, there is no experimental evidence of post-translational modifications or a contribution to parasitic adaptation at present, and further analyses are needed to determine the physiological function of PTORS.

A better understanding of host specificity would be obtained by analyses of host-parasite interactions, such as the mechanisms of evasion of the host defense system, adherence and/or invasion of host cells and tissue, and acquisition of nutrients from the host. Collectively, this study revealed common molecular signatures among mammalian *Entamoeba* species and an *E*. *nuttalli*-specific surface protein, based on refined assembly of the *E*. *nuttalli* genome and comparative genome analysis. The discovery of PTORS from *E*. *nuttalli* supports the validity of our catalog of molecular candidates related to host range. Our approach of host-driven comparative analysis of parasite molecules reflecting host specificity may be useful for prediction of possible host alternation, as well as understanding of parasite evolution and identification of new drug targets.

## Supporting information

S1 Fig Distribution of PacBio subreads length. Filter-passed subreads were obtained from 57 SMRT Cells of raw data with automatic removal of subreads with low accuracy (< 80%) and/or short-read length (< 500 bases). A bar plot was constructed using R with the bin width set to 1.
(TIF)

S2 Fig Efficiency of trimming of contig ends. After Illumina short-read data were mapped to the primary assembly and both contig ends of the primary assembly were trimmed, the mapped reads were counted at each position for each contig and aggregated per percentage of contig bases. The line plots indicate trimming of both contig ends before (upper) and after (lower).
(TIF)

S3 Fig Distribution of Gene Ontology function of *E*. *nuttalli*-specific candidate genes. Of 9,647 annotated genes derived from the *E*. *nuttalli* genome, 114 had weak matches against a public database and were only annotated in the InterPro collection database. Gene Ontology assignments of these 114 *E*. *nuttalli*-specific candidate genes were referred from the InterPro annotation using the interpro2go dataset.
(TIF)

S4 Fig Conservation of SALIP6 in *E*. *nuttalli* and *E*. *histolytica*. (A) ClustalW alignment of SALIP6 in *E*. *nuttalli* (EN0144G0007) and *E*. *histolytica* (EAL50434.1). The amino acid sequence of EN0144G0007 was classified into a protein signature by InterPro. Signal peptide and saposin-like (IPR011001) regions are framed by green and orange rectangles, respectively. (B) SignalP 4.1 analysis of EN0144G0007.
(TIF)

S5 Fig Phylogenetic tree for EN0096G0007 and its homologs in *Entamoeba* species. Putative homologous genes were collected from AmoebaDB using a BLAST search based on alignment score, e-value and visual inspection of the sequence alignment. Additionally, the putative homologous genes of *E*. *histolytica* were searched against our *E*. *nuttalli* protein dataset because some putative homologous genes of *E*. *nuttalli* could not be identified in AmoebaDB. Multiple alignments of the sequences were obtained using MAFFT v.7 with the G-INS-i algorithm and without gap region realignment (“Leave gappy regions”) (Katoh K, Standley DM. MAFFT multiple sequence alignment software version 7: improvements in performance and usability. Mol Biol Evol. 2013;30: 772–780.). The appropriate amino acid substitution model (JTT+G) for the reconstruction was selected using the “Find Best DNA/Protein Models” tool in MEGA7 (Kumar S, Stecher G, Tamura K. MEGA7: molecular evolutionary genetics analysis version 7.0 for bigger datasets. Mol Biol Evol. 2016;33: 1870–1874.). Unambiguously aligned positions were used in the Neighbor-Joining (NJ) and Maximum Likelihood (ML) methods with 1,000 bootstrap replications in MEGA7. The parameter of Rates among Sites was set as Gamma distributed (G) and value as 13. For construction of ML phylogeny, the parameter “Initial Tree File” was set as the NJ phylogeny that we constructed. The output best tree was further edited using FigTree v,1.4.3 (https://github.com/rambaut/figtree/). The support values at the nodes represent bootstrap values. EN0096G0007 was expressed as red text.
(TIF)

S6 Fig Prediction of EN0096G007 secondary structure. The predicted amino acid sequence of EN0096G007 was subjected to secondary structure analysis in Jpred and PSIPRED. Predicted α-helices, β-strands, coil and disordered regions are indicated as H, E, C and D, respectively. A predicted signal peptide sequence (SignalP, red rectangle) and transmembrane helix (SOSUI and TMPred, blue rectangle) are also shown.
(TIF)

S7 Fig Localization of EN0096G0007 protein in other *Entamoeba* species. Immunofluorescence images of *E*. *nuttalli*, *E*. *dispar* SAW1734RclAR and *E*. *moshkovskii* Laredo using antisera specific for PTORS. Scale bar = 10 μm. *Entamoeba* species were stained by treatment with Triton X-100 and with antisera specific for PTORS. Merged bright field and fluorescence images are also shown (Merge). These images are approximately 0.9 μm in thickness per section.
(TIF)

S8 Fig Comparison of orthologous clusters coverage between the new genome assembly and the public genome assembly in *E*. *nuttalli*. Venn diagram showing orthologous cluster coverage between the new genome assembly (in this study) and the public genome assembly (AmoebaDB ver.4.0) in *E*. *nuttalli*. Numbers are estimated by the OrthoVenn server. The number of orthologous clusters is shown in bold. Numbers given in brackets indicate the number of genes, including the orthologous clusters. The new genome assembly and public genome assembly have 1,090 and 530 singletons, respectively.
(TIF)

S9 Fig Number of BspAs in pathogenic *Entamoeba* species and their host proteins. (A) Comparison of the number of gene candidates for BspA-like proteins with and without putative membrane association among pathogenic *Entamoeba* species. *Entamoeba* BspA-like proteins predicted to contain “LRR_5 family (PF13306)” by Pfam (El-Gebali S, Mistry J, Bateman A, Eddy SR, Luciani A, Potter SC, et al. The Pfam protein families database in 2019. Nucleic Acids Res. 2018;47: D427-D432.) were collected from AmoebaDB. *Entamoeba* BspA-like proteins with putative membrane association were extracted from *Entamoeba* BspA-like proteins using TMHMM for the transmembrane and/or GPS-Lipid (Xie Y, Zheng Y, Li H, Luo X, He Z, Cao S, et al. GPS-Lipid: a robust tool for the prediction of multiple lipid modification sites. Sci Rep. 2016;6: 28249.) and PrePS (Maurer-Stroh S, Eisenhaber F. Refinement and prediction of protein prenylation motifs. Genome Biol. 2005;6: R55.) for lipid modification. (B) Correlation between the number of *BspA* genes in pathogenic *Entamoeba* species and genes for host fibronectin. (C) Correlation between the number of *BspA* genes in pathogenic *Entamoeba* species and genes for host fibrinogen. Human (*Homo sapiens*), macaque (*Macaca fascicularis*), and snake (*Python bivittatus*) are hosts for *E*. *histolytica*, *E*. *nuttalli*, and *E*. *invaden*s, respectively. Host genes were extracted from Genome Data Viewer (https://www.ncbi.nlm.nih.gov/genome/gdv/).
(TIF)

S10 Fig Reconstruction of a phylogenetic tree for the *Entamoeba* AIG1 protein family. A multiple sequence alignment of *Entamoeba* AIG1 family proteins was obtained in MUSCLE (Edgar RC. MUSCLE: multiple sequence alignment with high accuracy and high throughput. Nucleic Acids Res. 2004;32: 1792–1797.) and corrected by manual inspection. With 59 proteins from 3 species (16, 15, and 28 from *E*. *nuttalli*, *E*. *histolytica*, and *E*. *dispar*, respectively), 201 aligned amino acid sites were used in the analysis. The maximum likelihood (ML) best tree inferred by the JTT+F model with four categories of among-site rate variation and the rate variation model allowed some sites to be evolutionarily invariable (+I, 2.7363% sites) in MEGA7. Bootstrap proportions in the ML method (100 replicates) are attached to the internal branches. Branches with <50% bootstrap support are unmarked. Alignments are available from the authors upon request.
(TIF)

S1 Table Summary of Illumina sequencing and mapping data. Raw read data were obtained from the Illumina GAIIx platform with a 114 bp paired-end module. For trimming of low quality bases, raw read data were adapted with FASTX-Toolkit using a minimum read length (-l) of 70 bases and a quality cutoff (-t) of 20. The quality-passed reads were mapped to the primary assembly using bwa-mem with default parameters.
(XLSX)

S2 Table OrthoVenn results for *E*. *nuttalli*, *E*. *histolytica* HM-1:IMSS, *E*. *dispar* SAW760 and *E*. *invadens* IP-1. Proteome data were downloaded from AmoebaDB v.4.0.
(XLSX)
