# Supplementary figures and images for: Whole genome sequencing of Entamoeba nuttalli reveals mammalian host-related molecular signatures and a novel octapeptide-repeat surface protein
Source: PLoS Negl Trop Dis. 2019 Dec 5;13(12):e0007923. doi: 10.1371/journal.pntd.0007923 (PMC6917348; doi:10.1371/journal.pntd.0007923)

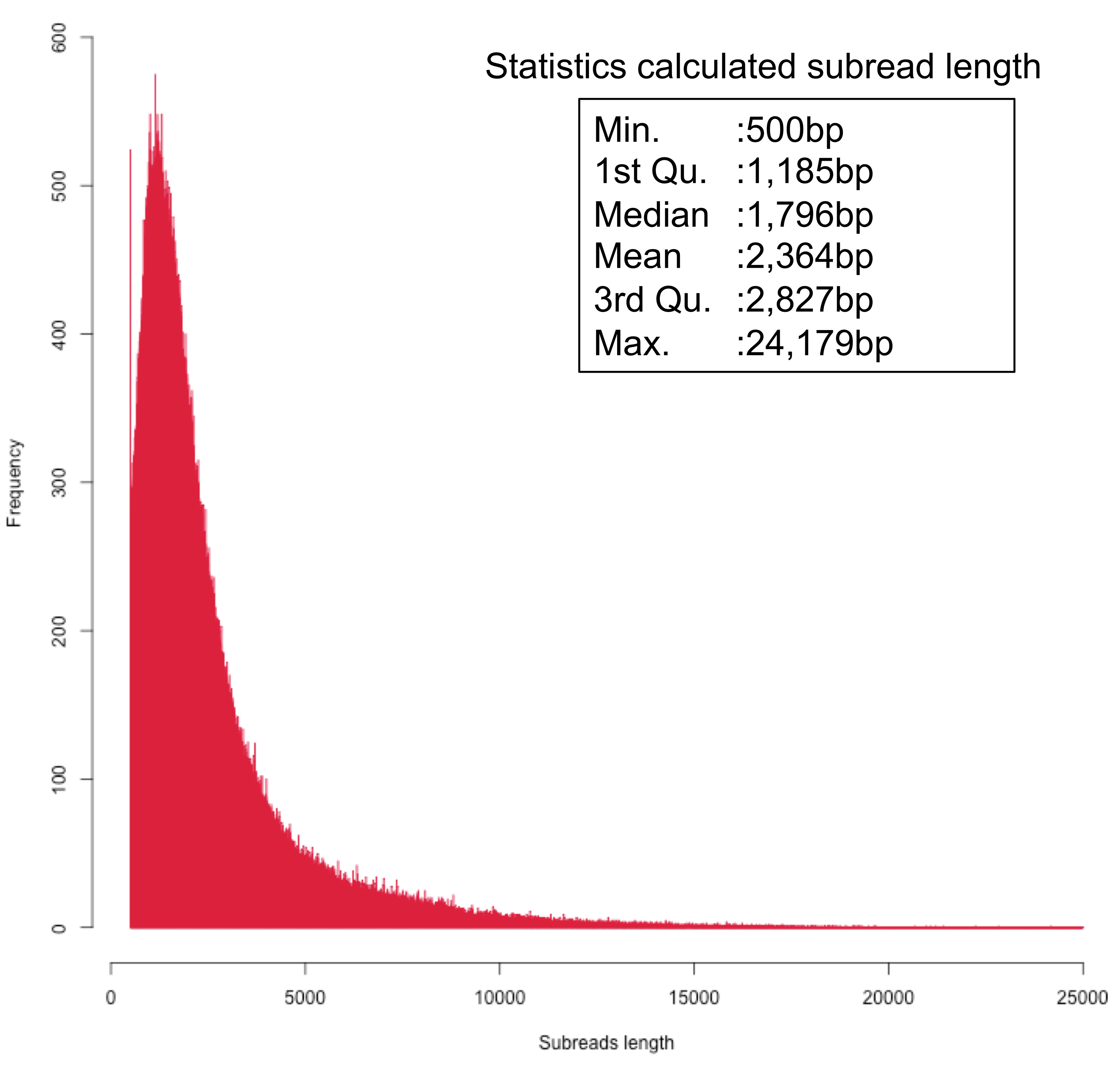

Supplement: S1 Fig — Filter-passed subreads were obtained from 57 SMRT Cells of raw data with automatic removal of subreads with low accuracy (< 80%) and/or short-read length (< 500 bases). A bar plot was constructed using R with the bin width set to 1. (TIF) [file pntd.0007923.s001.tif]

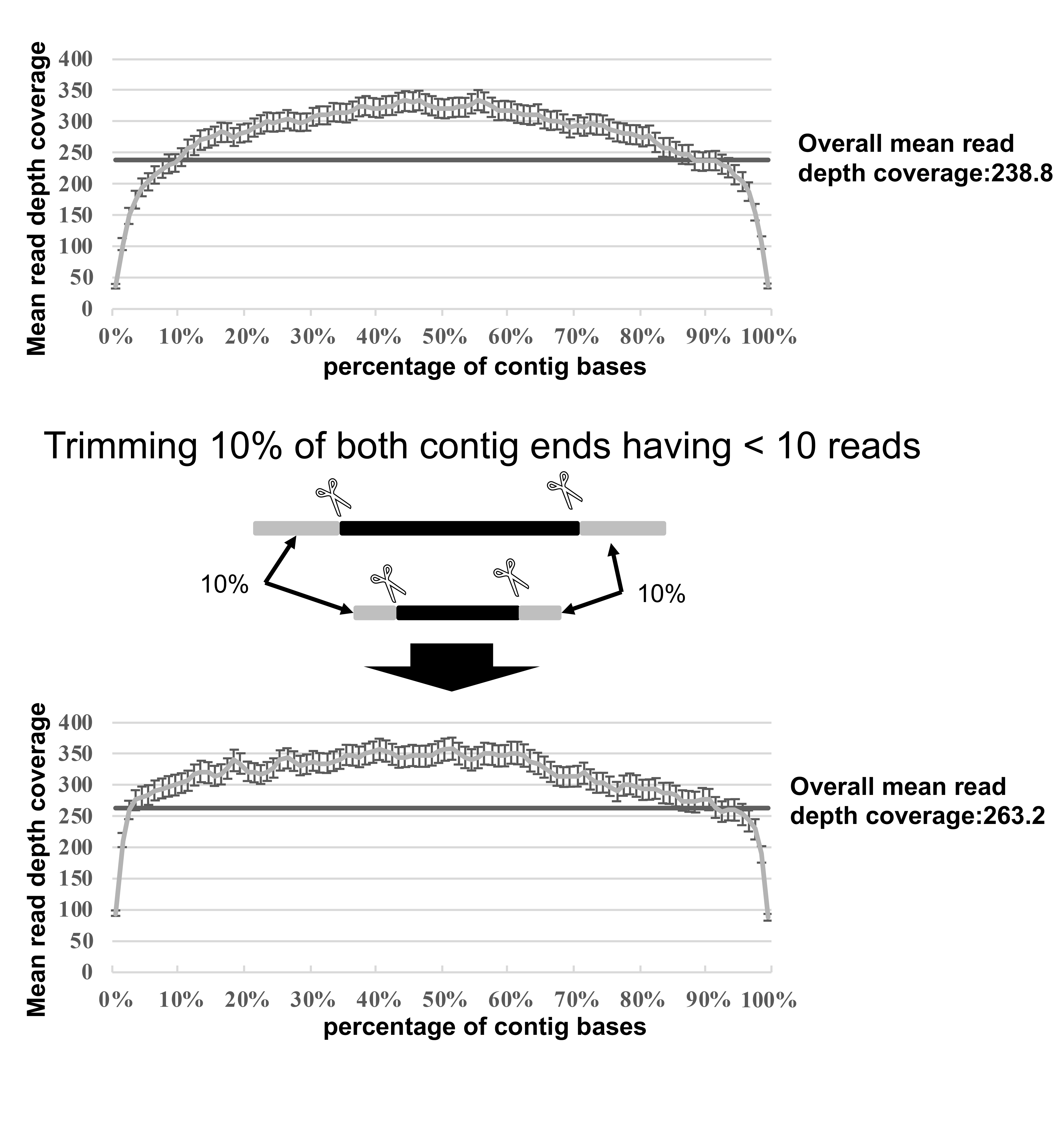

Supplement: S2 Fig — After Illumina short-read data were mapped to the primary assembly and both contig ends of the primary assembly were trimmed, the mapped reads were counted at each position for each contig and aggregated per percentage of contig bases. The line plots indicate trimming of both contig ends before (upper) and after (lower). (TIF) [file pntd.0007923.s002.tif]

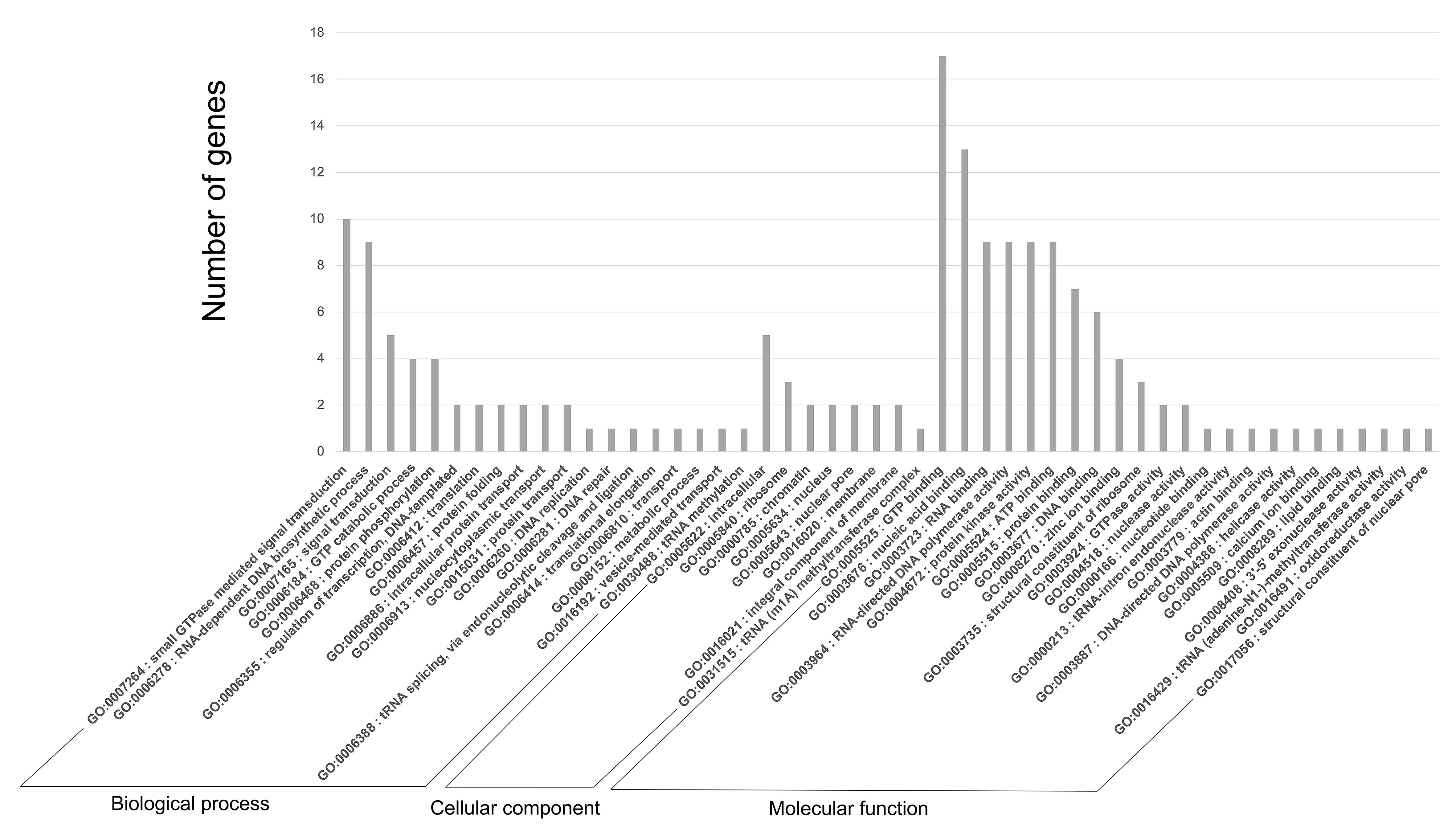

Supplement: S3 Fig — Of 9,647 annotated genes derived from the E. nuttalli genome, 114 had weak matches against a public database and were only annotated in the InterPro collection database. Gene Ontology assignments of these 114 E. nuttalli-specific candidate genes were referred from the InterPro annotation using the interpro2go dataset. (TIF) [file pntd.0007923.s003.tif]

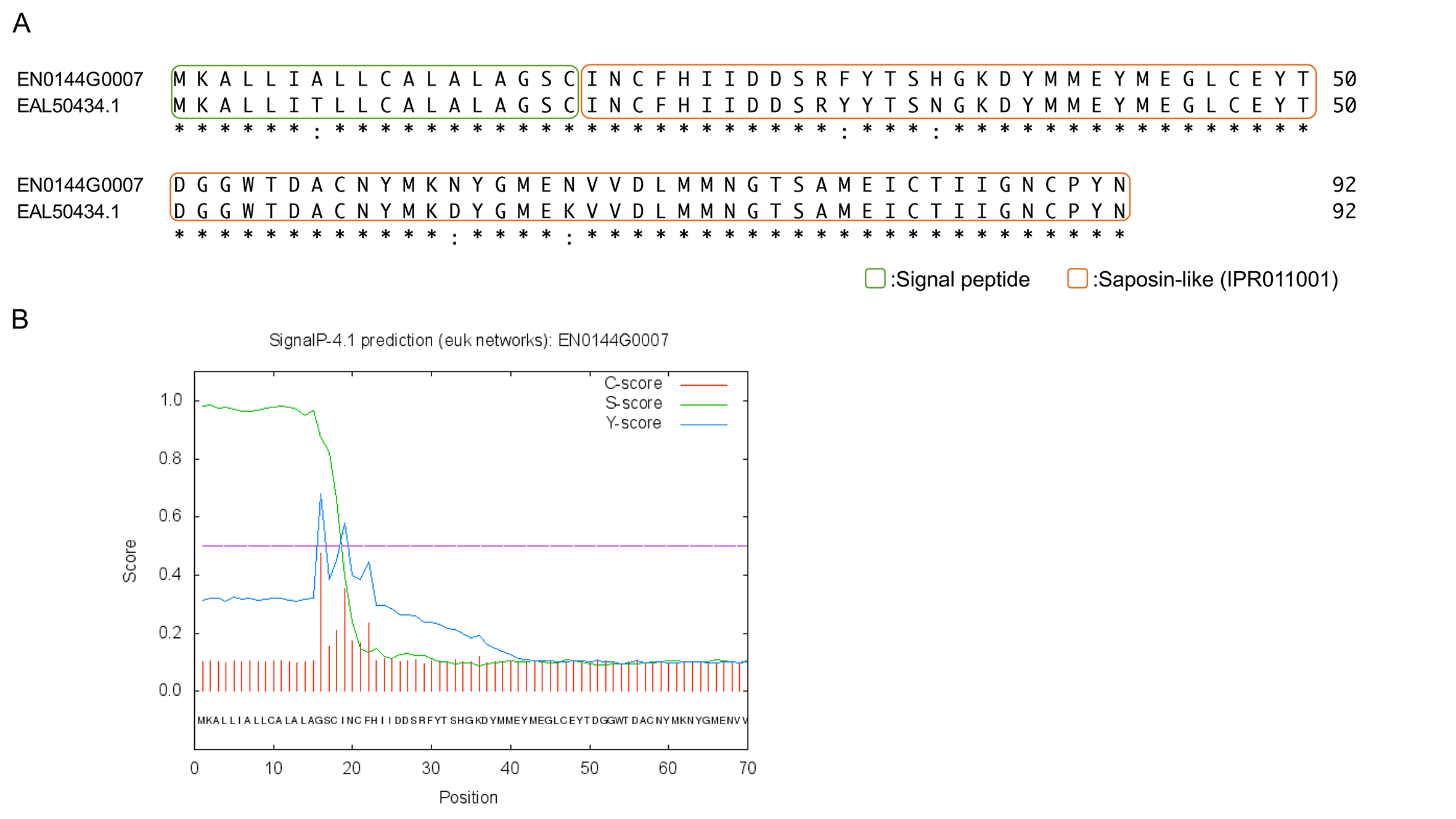

Supplement: S4 Fig — (A) ClustalW alignment of SALIP6 in E. nuttalli (EN0144G0007) and E. histolytica (EAL50434.1). The amino acid sequence of EN0144G0007 was classified into a protein signature by InterPro. Signal peptide and saposin-like (IPR011001) regions are framed by green and orange rectangles, respectively. (B) SignalP 4.1 analysis of EN0144G0007. (TIF) [file pntd.0007923.s004.tif]

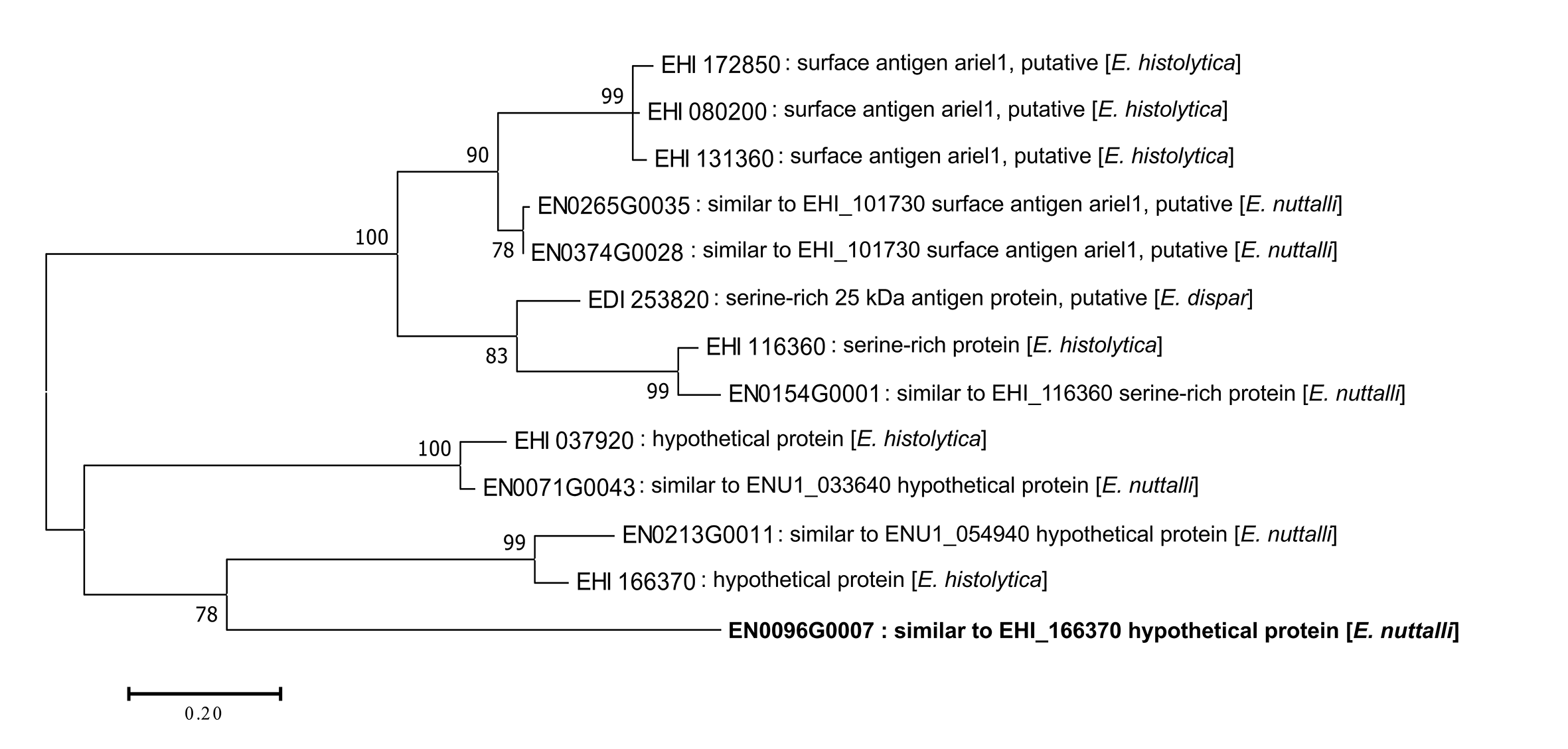

Supplement: S5 Fig — Putative homologous genes were collected from AmoebaDB using a BLAST search based on alignment score, e-value and visual inspection of the sequence alignment. Additionally, the putative homologous genes of E. histolytica were searched against our E. nuttalli protein dataset because some putative homologous genes of E. nuttalli could not be identified in AmoebaDB. Multiple alignments of the sequences were obtained using MAFFT v.7 with the G-INS-i algorithm and without gap region realignment (“Leave gappy regions”) (Katoh K, Standley DM. MAFFT multiple sequence alignment software version 7: improvements in performance and usability. Mol Biol Evol. 2013;30: 772–780.). The appropriate amino acid substitution model (JTT+G) for the reconstruction was selected using the “Find Best DNA/Protein Models” tool in MEGA7 (Kumar S, Stecher G, Tamura K. MEGA7: molecular evolutionary genetics analysis version 7.0 for bigger datasets. Mol Biol Evol. 2016;33: 1870–1874.). Unambiguously aligned positions were used in the Neighbor-Joining (NJ) and Maximum Likelihood (ML) methods with 1,000 bootstrap replications in MEGA7. The parameter of Rates among Sites was set as Gamma distributed (G) and value as 13. For construction of ML phylogeny, the parameter “Initial Tree File” was set as the NJ phylogeny that we constructed. The output best tree was further edited using FigTree v,1.4.3 (https://github.com/rambaut/figtree/). The support values at the nodes represent bootstrap values. EN0096G0007 was expressed as red text. (TIF) [file pntd.0007923.s005.tif]

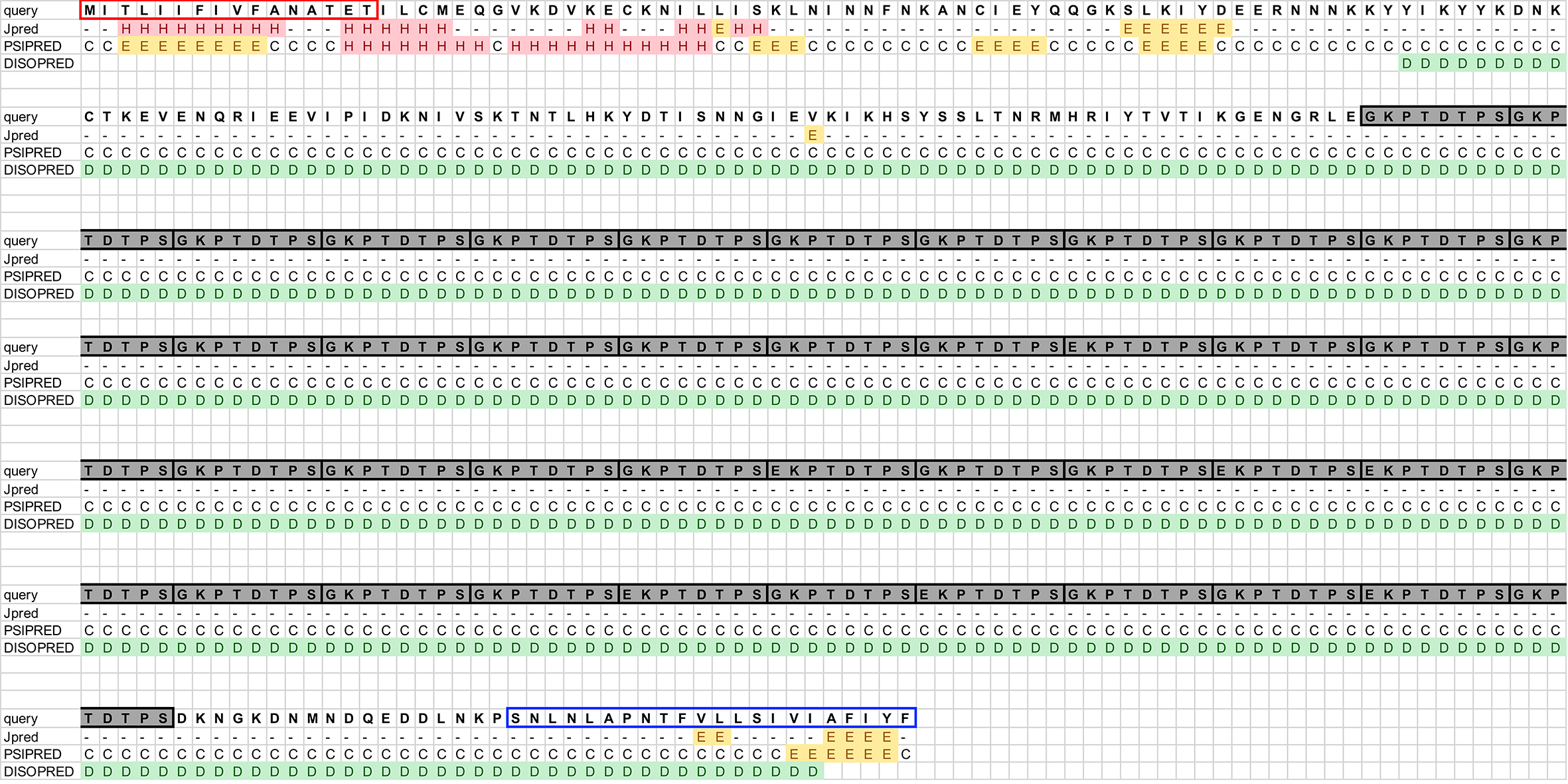

Supplement: S6 Fig — The predicted amino acid sequence of EN0096G007 was subjected to secondary structure analysis in Jpred and PSIPRED. Predicted α-helices, β-strands, coil and disordered regions are indicated as H, E, C and D, respectively. A predicted signal peptide sequence (SignalP, red rectangle) and transmembrane helix (SOSUI and TMPred, blue rectangle) are also shown. (TIF) [file pntd.0007923.s006.tif]

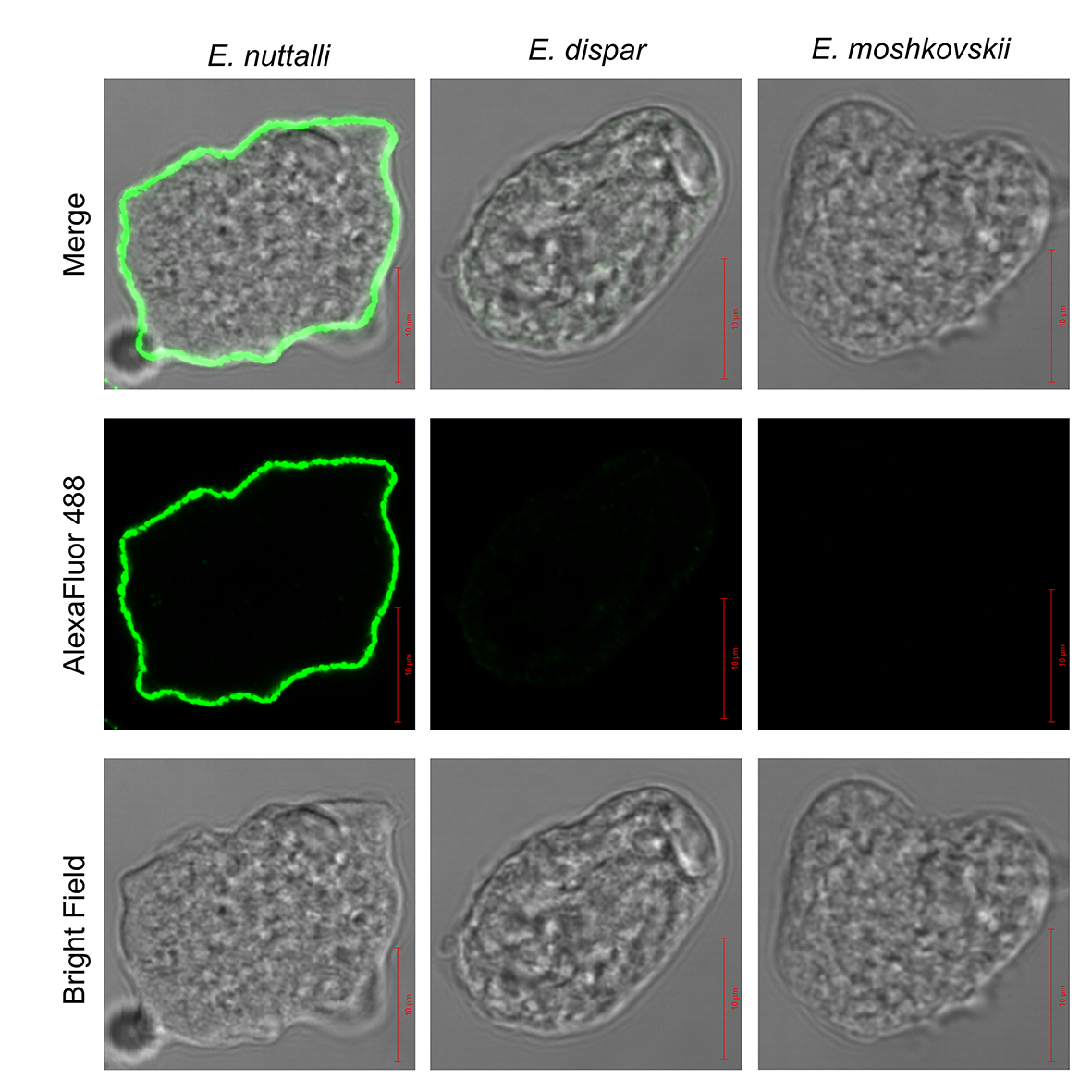

Supplement: S7 Fig — Immunofluorescence images of E. nuttalli, E. dispar SAW1734RclAR and E. moshkovskii Laredo using antisera specific for PTORS. Scale bar = 10 μm. Entamoeba species were stained by treatment with Triton X-100 and with antisera specific for PTORS. Merged bright field and fluorescence images are also shown (Merge). These images are approximately 0.9 μm in thickness per section. (TIF) [file pntd.0007923.s007.tif]

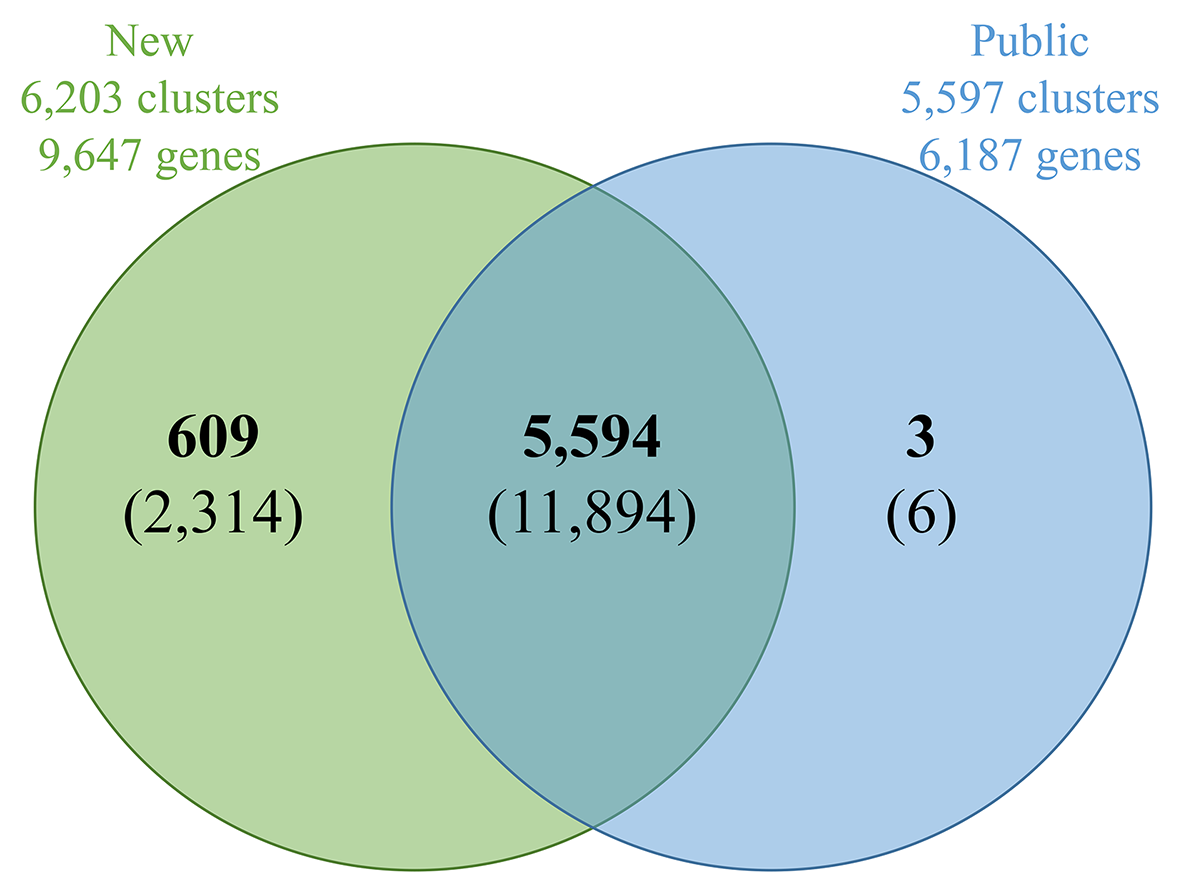

Supplement: S8 Fig — Venn diagram showing orthologous cluster coverage between the new genome assembly (in this study) and the public genome assembly (AmoebaDB ver.4.0) in E. nuttalli. Numbers are estimated by the OrthoVenn server. The number of orthologous clusters is shown in bold. Numbers given in brackets indicate the number of genes, including the orthologous clusters. The new genome assembly and public genome assembly have 1,090 and 530 singletons, respectively. (TIF) [file pntd.0007923.s008.tif]

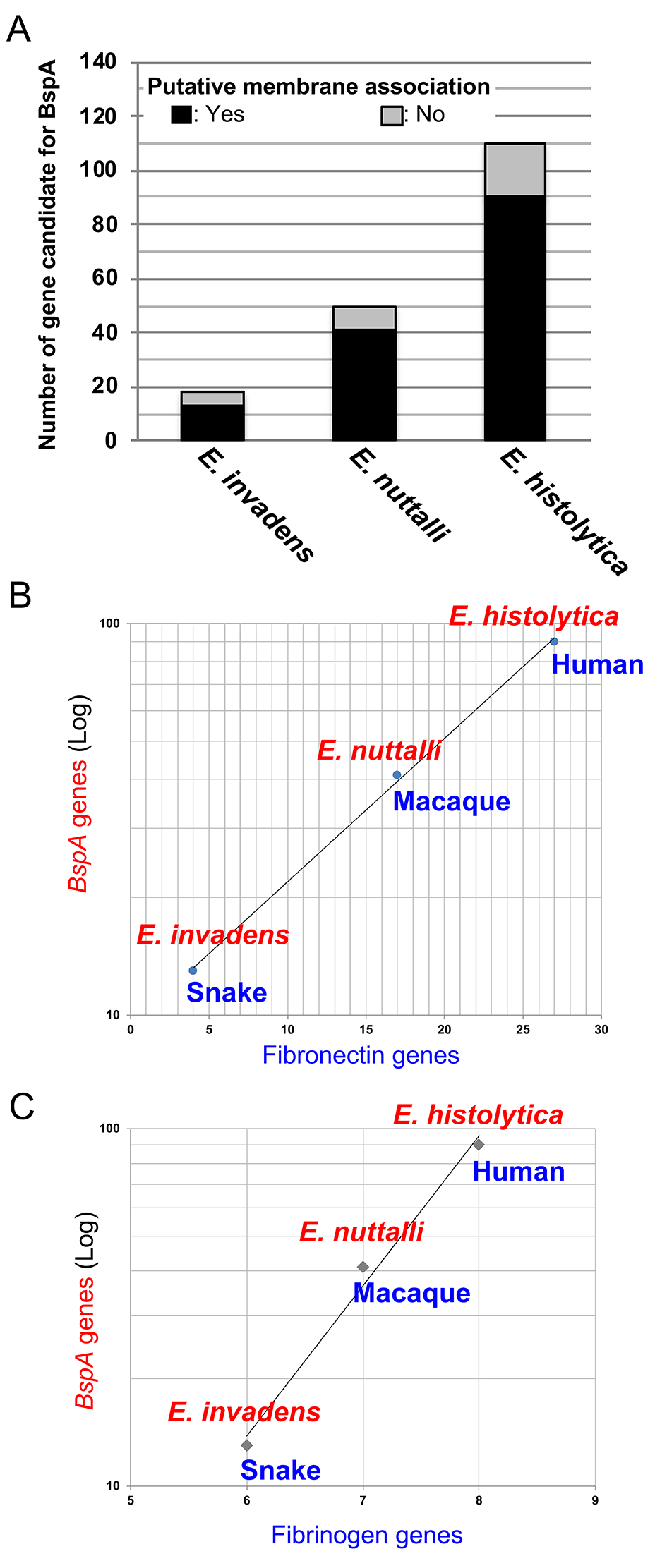

Supplement: S9 Fig — (A) Comparison of the number of gene candidates for BspA-like proteins with and without putative membrane association among pathogenic Entamoeba species. Entamoeba BspA-like proteins predicted to contain “LRR_5 family (PF13306)” by Pfam (El-Gebali S, Mistry J, Bateman A, Eddy SR, Luciani A, Potter SC, et al. The Pfam protein families database in 2019. Nucleic Acids Res. 2018;47: D427-D432.) were collected from AmoebaDB. Entamoeba BspA-like proteins with putative membrane association were extracted from Entamoeba BspA-like proteins using TMHMM for the transmembrane and/or GPS-Lipid (Xie Y, Zheng Y, Li H, Luo X, He Z, Cao S, et al. GPS-Lipid: a robust tool for the prediction of multiple lipid modification sites. Sci Rep. 2016;6: 28249.) and PrePS (Maurer-Stroh S, Eisenhaber F. Refinement and prediction of protein prenylation motifs. Genome Biol. 2005;6: R55.) for lipid modification. (B) Correlation between the number of BspA genes in pathogenic Entamoeba species and genes for host fibronectin. (C) Correlation between the number of BspA genes in pathogenic Entamoeba species and genes for host fibrinogen. Human (Homo sapiens), macaque (Macaca fascicularis), and snake (Python bivittatus) are hosts for E. histolytica, E. nuttalli, and E. invadens, respectively. Host genes were extracted from Genome Data Viewer (https://www.ncbi.nlm.nih.gov/genome/gdv/). (TIF) [file pntd.0007923.s009.tif]

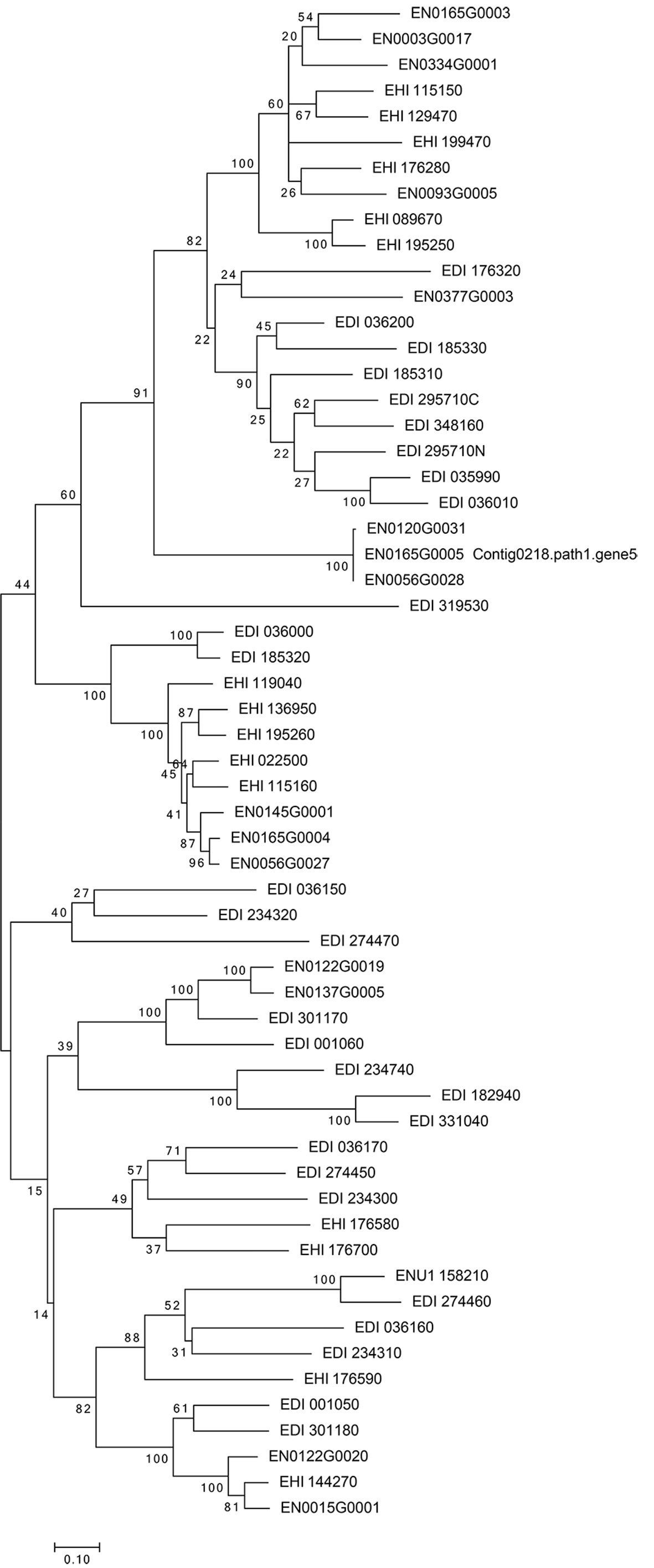

Supplement: S10 Fig — A multiple sequence alignment of Entamoeba AIG1 family proteins was obtained in MUSCLE (Edgar RC. MUSCLE: multiple sequence alignment with high accuracy and high throughput. Nucleic Acids Res. 2004;32: 1792–1797.) and corrected by manual inspection. With 59 proteins from 3 species (16, 15, and 28 from E. nuttalli, E. histolytica, and E. dispar, respectively), 201 aligned amino acid sites were used in the analysis. The maximum likelihood (ML) best tree inferred by the JTT+F model with four categories of among-site rate variation and the rate variation model allowed some sites to be evolutionarily invariable (+I, 2.7363% sites) in MEGA7. Bootstrap proportions in the ML method (100 replicates) are attached to the internal branches. Branches with <50% bootstrap support are unmarked. Alignments are available from the authors upon request. (TIF) [file pntd.0007923.s010.tif]
